# Reimagining reproductive futures: Speculative Design as an Artistic-Public Thought Experiment

**DOI:** 10.1038/s44319-026-00897-z

**Published:** 2026-08-05

**Authors:** Bridget X Li, Aviad Raz, Jusaku Minari

**Affiliations:** 1 Department of Biology, Massachusetts Institute of Technology, Cambridge, MA USA; 2 Department of Sociology and Anthropology, Ben-Gurion University of the Negev, Beer-Sheba, Israel; 3 Uehiro Research Division for iPS Cell Ethics, Center for iPS Cell Research and Application, Kyoto University, Kyoto, Japan

**Keywords:** History & Philosophy of Science, Methods & Resources

## Abstract

This article discusses speculative design as a creative practice that uses imagined socio-technological scenarios to provoke debate and reflection in the context of reproductive technologies.

**Figure:**
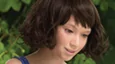

Biomedical sciences and technologies—from artificial reproductive technologies (ART) to stem-cell-based tissue and organ synthesis to genome editing—consistently expand the boundaries of what is considered technically and medically possible. However, public awareness of and perspectives on possible future applications often struggle to keep pace as these technologies evolve. This gap raises fundamental questions about how experts and nonexperts consider and negotiate the social, moral, and normative implications of scientific advancements and biomedical technologies. Within this context, speculative design (SD) has emerged as a distinctive mode of public engagement that draws on ambiguity and dissonance, thereby functioning as a thought experiment that can surface underlying assumptions and stimulate reflection (Dunne and Raby, 2013; Cardenas Cordova et al, 2025). SD differs not only from conventional or dominant forms of public engagement, which aim to make science more familiar or accessible to the public, but also from art-based science communication, which often uses art to communicate scientific knowledge (Mierzwa and Goodsell, 2021; Okete, 2024), as well as from understandings of artistic practice as a site of knowledge production and critical inquiry in its own right (Anker, 2021). We argue that SD occupies a “third space” in science–art collaboration (Groves et al, 2025), which is not reducible to either science communication or ELSI/bioethics. It offers a conceptual lens through which the intersection of science and society can be explored as a site of critical reflection and imaginative inquiry (Bulani, 2022; Cardenas Cordova et al, 2025).

… SD occupies a “third space” in science–art collaboration […], which is not reducible to either science communication or ELSI/bioethics.

In this commentary, we use the example of ART to highlight how SD mediates engagement between science and society. Building on the theoretical foundations established by critical design theorists Anthony Dunne and Fiona Raby (2013), we demonstrate how SD constructs affective and conceptual bridges that enable both nonexperts and experts to engage with and critically reflect on the moral and political stakes of emerging reproductive technologies, even when such developments unfold beyond public awareness. Through two illustrative examples, we show how SD can reveal underlying normative assumptions, foster inclusive deliberation, and extend public ethical imagination beyond conventional science communication and bioethics. SD is not only a theoretical framework but also a practical means of facilitating critical reflection and public ethical reasoning. While our discussion centers on ART, the argument extends to how SD can more broadly contribute to reimagining the relations between science and society.

## The role of speculative design in bringing together science and society

SD emerged from the broader tradition of critical design articulated by Dunne and Raby (2013), who argue that design can serve not only commercial or functional purposes but also critical and conceptual ones. From this perspective, we conceptualize SD as a mode of making the “not yet” visible by designing plausible and implausible futures. Through this process, it invites the public to reflect on how scientific trajectories intersect with cultural norms, political aspirations, and social anxieties. In the context of reproductive technologies, SD positions ART not as a clinical procedure but as a site where future kinship structures, gender norms, and family imaginaries are envisaged and contested.

In the context of reproductive technologies, SD positions ART not as a clinical procedure but as a site where future kinship structures, gender norms, and family imaginaries are envisaged and contested.

**Fig. 1:**
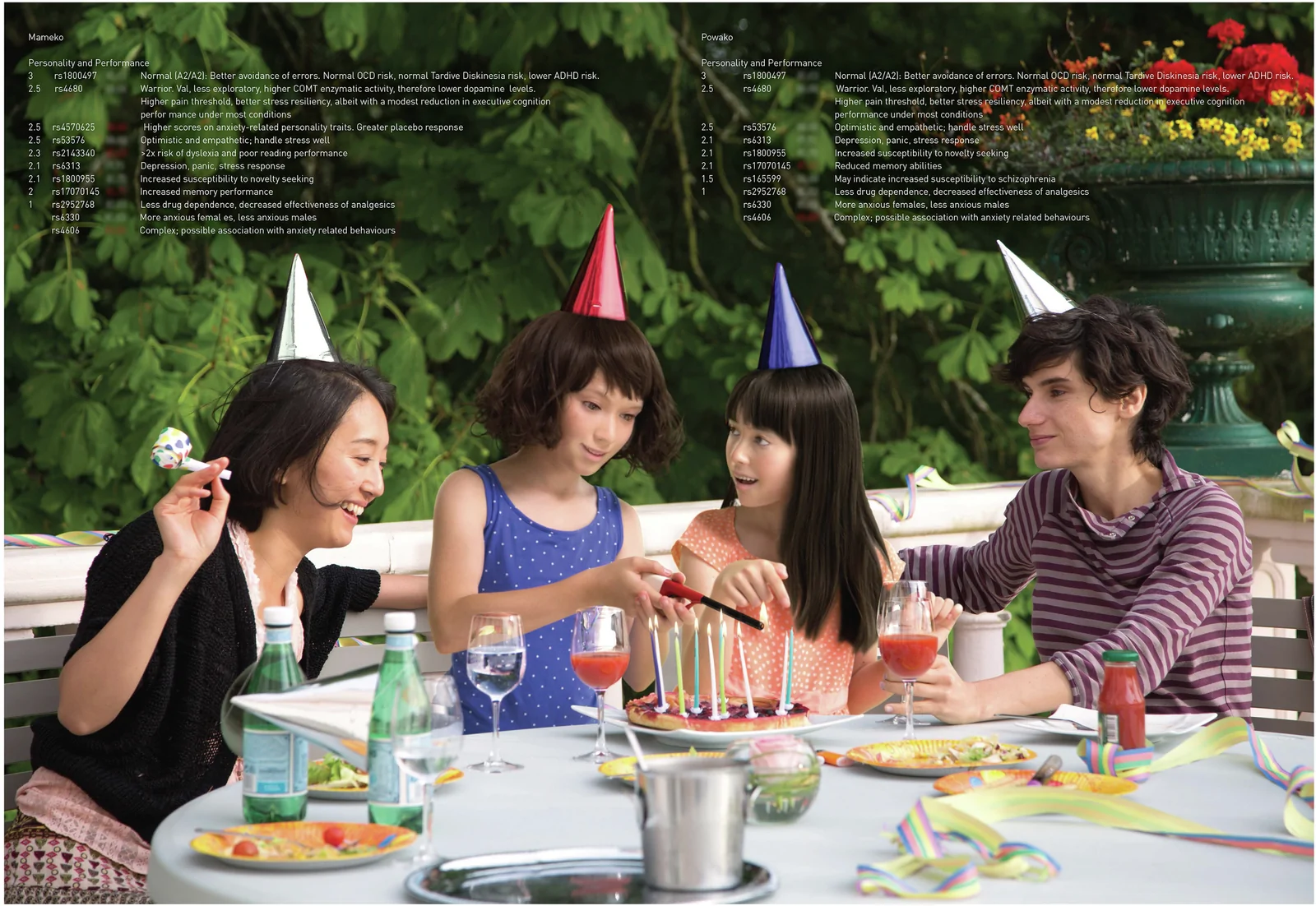
Image from “(Im)possible Baby”, Case 01: Asako and Moriga by Ai Hasegawa (2015). With permission from the artist.

Ai Hasegawa’s project *(Im)possible Baby, Case 01: Asako and Moriga* (for short, *(Im)possible Baby*) visualizes a future in which two cisgender women have a genetically related child using technologies such as stem-cell-derived gametes. Rather than focusing on the laboratory details or the science, Hasegawa situates this speculative family in everyday settings: breakfasts, birthday celebrations, and domestic routines, thereby producing cognitive dissonance (Fig. 1). Viewers encounter a family that feels emotionally familiar, yet its genetic and biological underpinnings—which are also detailed as part of the project—are speculative. This intentional juxtaposition reveals that ideas of kinship are both biologically grounded and culturally constructed.

By creating intimate visual narratives around speculative reproduction, the artist enables broader publics to engage with the moral and political stakes of genetic parenthood, queer reproduction, and the role of biotechnology. Hasegawa, who created this award-winning graduate thesis project at the MIT Media Lab in 2015, commented on the importance of her SD project as a facilitator of public engagement: “to achieve more public outreach, I worked with the Japanese national television NHK to create a 30-min documentary film which documented the whole process of analyzing the DNA and designing the photo album. The film also included our interviews with scientists and the lesbian couple about their views on bioethics. Finally, the project will analyze and evaluate the public reaction triggered by this documentary program after it is aired nationally in Japan, and also after it is subtitled and released online, available to more people internationally.”

**Fig. 2:**
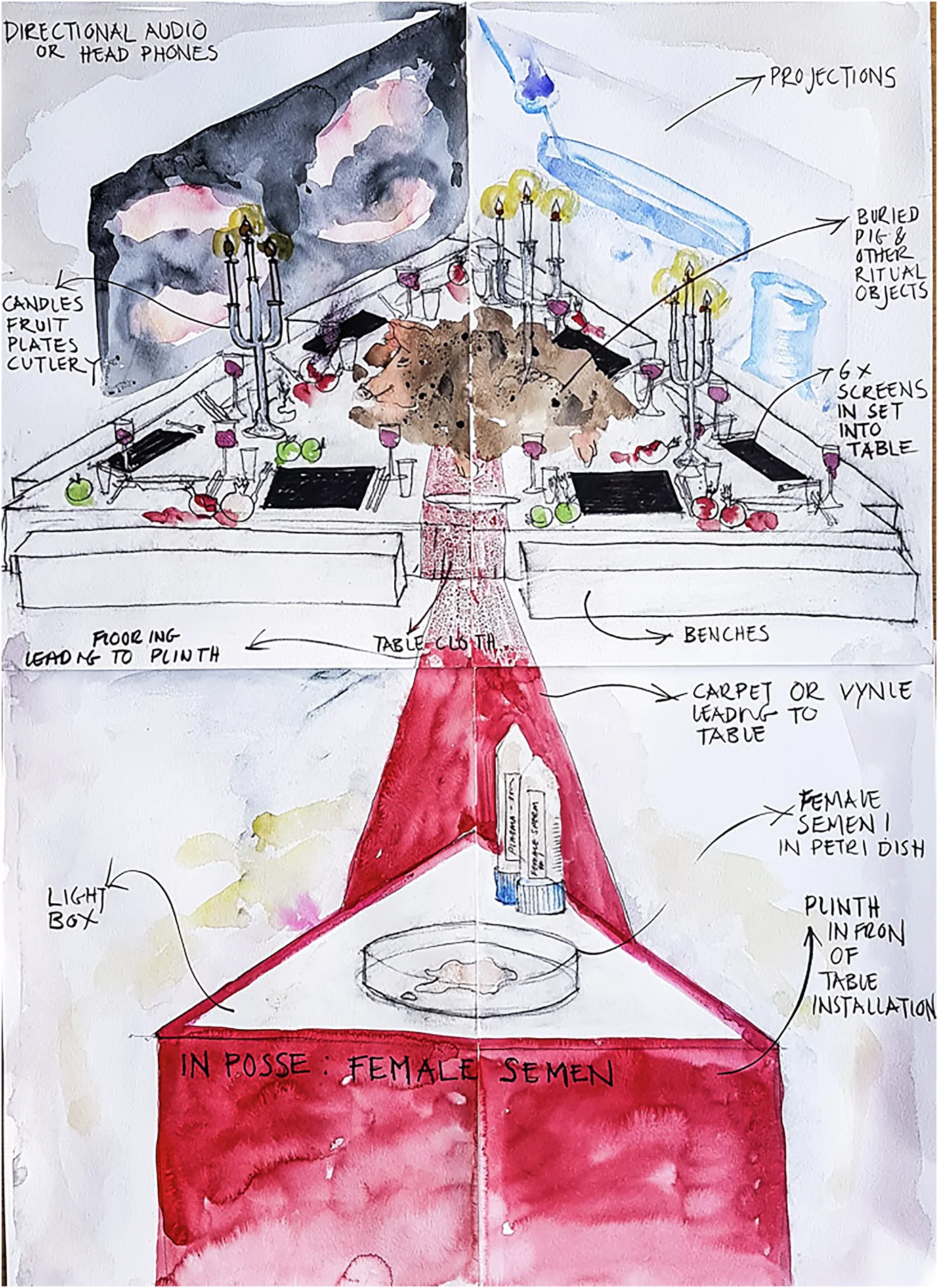
Installation sketch for “In Posse” exhibit at Site Gallery, Sheffield, by Charlotte Jarvis (2021). With permission from the artist.

British artist Charlotte Jarvis’s *In Posse*—Latin for “in possibility” or “pre-born”—adopts a different design approach. Rather than visualizing hypothetical futures, Jarvis engages with scientific collaborators to explore whether differentiating stem cells from her and other contributors into sperm-like cells could produce “female semen.” This SD project, which began in 2019, with exhibitions and developments continuing until 2021 in the Netherlands, the UK, and Slovenia, is explicitly political, challenging patriarchal assumptions about biological roles and reproduction. Unlike Hasegawa’s domestic familiarity, Jarvis foregrounds the materiality of scientific processes—cell cultures, laboratory techniques and genetic modification—to provoke questions about autonomy and power, reproductive rights, the division of reproductive labor and the possibilities of biotechnology (Fig. 2). This SD project was designed to be highly participatory, engaging the public through blood plasma donation, participatory re-enactments of the ancient women-only Greek Festival of Thesmophoria and a film showcasing the project, which has been presented in panel discussions, workshops and exhibitions to prompt public debate.

Both projects demonstrate SD as a mediator between scientific research and the public imagination by engaging with ART in distinct ways. Hasegawa renders these possibilities affectively accessible through familiar scenes, whereas Jarvis foregrounds the material and political implications through direct engagement with scientific processes. Despite these differences in approach, by constructing tangible or visual scenarios, SD enables audiences to confront questions that often remain abstract in scientific discourse: What makes a family feel “real?” Who is permitted to reproduce? How do gender norms shape biological expectations, and how might reproductive technologies enforce, challenge, or transform these structures?

More broadly, SD complements and extends existing models of public communication. Traditional science communication generally focuses on conveying accurate information, while deliberative public engagement frameworks prioritize dialog, structured reflection, and policy-oriented foresight. SD, by contrast, operates through ambiguity, dissonance, and affect, allowing publics to inhabit future scenarios without requiring scientific literacy or policy expertise. Building on Tsekleves et al (2022), we argue that SD contributes a crucial missing layer to the science–society interface by fostering emotional, imaginative, and experiential engagement with emerging biotechnologies.

… SD contributes a crucial missing layer to the science–society interface by fostering emotional, imaginative, and experiential engagement with emerging biotechnologies.

## Situating speculative design within science and society interventions

We therefore argue that SD is a distinct mode of public thought experimentation that expands the repertoire of tools for engaging with the public beyond classical science communication, public outreach, or, specifically, art. Zhu and Goyal (2019) argue that art and science share common foundations in observation, interpretation, and representation of nature and that both contribute to ways of depicting natural phenomena and communicating ideas. Similarly, Frazzetto (2004) highlights the increasing intersections between science and art through collaborative projects that engage with contemporary scientific practices and their social implications. Artistic representation of science is thereby a vehicle for science dissemination and a mirror of society’s responses to science and technology.

Our argument extends this reasoning by showing that SD can enact such engagements in ways that are not readily achievable in conventional art-science and science communication practices. Rather than asking the public to articulate positions within structured settings, SD enables them to encounter futures viscerally, sometimes even before they form clear opinions. Furthermore, SD does not merely interpret science; it stages alternative worlds for audiences to inhabit, thereby generating new conceptual frameworks for thinking about scientific areas such as reproduction, kinship, and technology. By creating counterfactual scenarios, SD becomes not only reflective but also generative.

While art can be more effective in offering people direct experience of phenomena compared with simply reading explanations or viewing charts, graphs, or data, it is “there to make sense of what they [scientists] are doing, to make sense of what the outcome of research actually means for society and to raise questions” (King, 2019). Building on this characterization, we argue that SD contributes to a distinct, complementary form of anticipatory governance. Whereas governance frameworks often emphasize prediction, risk assessment, or ethical analysis, SD foregrounds possibility, plurality, and the emotional dimensions of technological progress.

## Speculative design as a means to facilitate public thought experiments

SD is most impactful when it challenges entrenched assumptions. To do so effectively, designers must be clear about the intentions, philosophical foundations, and methodological practices that differentiate SD from other forms of artistic or scientific exploration.

SD is most impactful when it challenges entrenched assumptions.

One way to sharpen this distinction is to frame SD as a form of public thought experiment. Drawing on recent literature on thought experiments (Stuart et al, 2018), SD can be understood as a means of testing intuitions, exposing conceptual blind spots, and examining value conflicts through artificially constructed scenarios. While traditional thought experiments in science or philosophy rely on imagined conditions, such as Schrödinger’s cat and Thomson’s violinist, SD translates these abstract exercises into material, visual, and experiential forms, making public participation more accessible and inviting interpretive engagement.

This philosophical orientation distinguishes SD from art and design. While art often seeks to produce aesthetic experience and design aims to develop functional solutions to defined problems, SD is primarily concerned with conceptual inquiry rather than beauty or utility. SD projects are deliberately unfinished, provocative or exaggerated, encouraging viewers not to accept predefined answers but instead to formulate their own questions (Dunne and Raby, 2013). Ambiguity and critical uncertainty lie at the core of this approach. To strengthen SD as a medium for public engagement, we propose four interrelated directions, with attention to contexts including ART. Together, these directions reposition SD as a practice that operates across epistemic, methodological, normative, and experiential dimensions.

First, SD should foreground scientific uncertainty more explicitly. Rather than presenting imagined futures as inevitable extensions of current trajectories, it can make visible the gaps between scientific capability and speculative extrapolation. This framing enables audiences to distinguish more clearly between what is possible and what remains unknown, contested or implausible.

Second, SD can benefit from participatory methodologies. As illustrated by Jarvis’s *In Posse* project, media events, workshops, scenario-building sessions, and collaborative prototyping with diverse publics—including queer families, patients undergoing fertility treatment, clinicians, and scientists—can help ensure that speculative futures reflect a wider array of lived experiences and values. In this sense, participatory SD transforms speculation from a unidirectional provocation into a collective process of imagination.

Third, SD can more explicitly surface embedded value systems. Rather than treating future reproductive possibilities as simply extensions of existing technological trajectories, it can interrogate the normative assumptions behind these systems: Why is genetic relatedness valued? Why do certain forms of reproductive labor remain feminized? How do sociotechnical imaginaries shape what futures are considered desirable or undesirable?

Fourth, SD can adopt exhibition strategies that promote sustained engagement. Given that museum visitors typically spend limited time with individual installations, compact and interconnected exhibits may guide audiences through speculative scenarios and encourage deeper reflection (Serrell, 1997). By structuring exhibitions as sequences of thought-provoking vignettes, SD can scaffold public engagement over time, allowing interpretive meaning to unfold gradually.

Ultimately, SD is most effective when it bridges incredulity and engagement—when it moves viewers from initial surprise toward deeper reflection. As recent empirical work demonstrates, SD can illuminate the values underlying concepts such as “naturalness” or “normality” in reproductive medicine (Willems et al, 2023). By presenting unfamiliar futures in accessible, experiential formats, it opens spaces for public deliberation that are not easily cultivated by traditional science communication or policy consultation.

## Conclusion

In this commentary, we advance the conceptual claim that SD can connect science and society, positioning it not as “art about science” but as a means of articulating imagined futures. SD is not merely artistic visualization but also functions like a thought experiment, a philosophical technology for exploring counterfactuals. We argue that SD is therefore novel in two ways. First, as a “third mode” of public engagement—beyond ELSI (which foregrounds ethical, legal and social analysis) and science communication (which informs the public)—it operates as a participatory imaginary practice that blends affect, aesthetics and moral reflection. Second, it exposes the normative scaffolding behind emerging possibilities, such as kinship, gender roles, genetic inheritance, and bodily autonomy in reproductive biotechnology.

By expanding and building on other modalities—for instance, ELSI research, public engagement exercises, and bioethics—SD mediates between communities: scientists and stem cell/ART researchers; publics and patient groups; policymakers and constituencies confronting the future of reproduction. Moreover, it does not predict the future of reproduction but democratizes who gets to imagine it.

In these examples, SD occupies the space between imagination and scientific possibility. In the domain of reproductive technology, it offers a unique form of public thought experimentation that brings scientific trajectories into dialog with cultural norms, personal identities, and political visions. By visualizing future kinship structures, prototyping alternative reproductive capacities, and staging encounters with speculative biologies, SD renders the act of imagining reproductive futures as potentially inclusive.

At the same time, SD must confront its limitations. Its scenarios can reproduce the exact normative assumptions that they seek to challenge. Its audiences may misunderstand or dismiss speculative works if the imagined futures feel too abstract or implausible. In addition, its impact depends on the design of exhibitions, participatory engagements, and collaborative processes that make speculative scenarios accessible and meaningful. Nevertheless, these challenges are also opportunities. By embracing transparency, participation, and philosophical rigor, SD can evolve into a powerful tool for responsible research and innovation and complement scientific practice by illuminating the social and ethical dimensions of emerging technologies and policy frameworks.

SD can evolve into a powerful tool for responsible research and innovation and complement scientific practice by illuminating the social and ethical dimensions of emerging technologies and policy frameworks.

SD does not predict what is to come. Instead, it expands the terrain of what can be collectively imagined, questioned, and negotiated. As reproductive technologies continue to advance, SD invites us to think within and through possible futures, ensuring that the development of biotechnology remains accountable not only to scientific progress but also to human values, lived experiences, and shared visions of a just and inclusive society.

## Supplementary information

Peer Review File
