# Supplementary material for: Reimagining reproductive futures: Speculative Design as an Artistic-Public Thought Experiment
Source: EMBO Rep. 2026 Aug 5;27(17):4940–4. doi: 10.1038/s44319-026-00897-z (PMC13554213; doi:10.1038/s44319-026-00897-z)
Supplement: Supplementary file 1 [file 44319_2026_897_MOESM1_ESM.pdf]

EMBO reports

Reimagining Reproductive Futures: Speculative Design as an Artistic-Public Thought Experiment

Bridget Li, Aviad Raz, and Jusaku Minari

Corresponding author(s): Jusaku Minari (jusaku.minari@cira.kyoto-u.ac.jp)

**Review Timeline:**	Submission Date:	3rd May 26
	Accepted:	29th Jul 26

Editor: Holger Breithaupt

Transaction Report: This article was editorially reviewed.
